# KDR Mutation as a Novel Predictive Biomarker of Exceptional Response to Regorafenib in Metastatic Colorectal Cancer

**DOI:** 10.7759/cureus.478

**Published:** 2016-02-03

**Authors:** Arturo Loaiza-Bonilla, Christopher E Jensen, Stuti Shroff, Emma Furth, Paula A Bonilla-Reyes, Andres F Deik, Jennifer Morrissette

**Affiliations:** 1 Medicine, Hematology and Oncology, Abramson Cancer Center of the University of Pennsylvania; 2 Department of Medicine, The Hospital of the University of Pennsylvania; 3 Department of Pathology and Laboratory Medicine, University of Pennsylvania School of Medicine, Philadelphia, PA; 4 Facultad de Medicina, Pontificia Universidad Javeriana; 5 Department of Neurology, University of Pennsylvania School of Medicine, Philadelphia, PA

**Keywords:** colorectal cancer, exceptional responder, genomic medicine, kdr, molecular tumor board, next-generation sequencing, personalized medicine, regorafenib, tki, vegfr-2

## Abstract

This is the case of an 84-year-old woman diagnosed with Stage IVb colon adenocarcinoma (CRC) metastatic to the liver, retroperitoneum, anastomotic site, and distal rectal sigmoid colon. She experienced intolerable side effects to systemic chemotherapy with 5-fluorouracil and bevacizumab, as well as disease progression. Next generation sequencing of her tumor was ordered, and further discussion of her malignancy’s genomic information took place at a multidisciplinary molecular tumor board. The patient had mutations in KRAS (Kirsten rat sarcoma viral oncogene homolog) which made her ineligible for epidermal growth factor receptor (EGFR) inhibitors; however, a KDR p.R961W c.2881C>T mutation was noted as a variant of unknown significance (VUS). KDR (kinase insert domain receptor) is the human gene encoding for vascular endothelial growth factor receptor 2 (VEGFR-2). She was then considered a suitable candidate for regorafenib, which she could only tolerate at a low dose of 40 mg daily, with the intent of prolonging her survival and to optimize her quality of life. We report her excellent tolerance and exceptional response to low dose regorafenib, including symptomatic, tumor marker, and sustained partial metabolic radiological improvement. In the largest Phase III trial of regorafenib in CRC, only five patients (1%) of 760 experienced a partial response (versus one patient, 0.4%, receiving placebo). KDR R961W mutation has been described but no functional data has been reported. This mutation occurs in the tyrosine kinase domain of the VEGFR-2. Regorafenib targets VEGFR-2 (KDR). Hereby we hypothesize KDR mutation as a novel predictive biomarker to exceptional response to regorafenib in metastatic colorectal cancer. To our knowledge, this is the first reported case of the potential correlation between KDR mutation and regorafenib use for the successful management of a patient with advanced CRC, leading to what is considered an exceptional response. Further studies based on this preliminary data are warranted.

## Introduction

Colorectal cancer (CRC) is the third most common cause of cancer death worldwide, representing about 10% of cancer diagnoses and mortality, with more than 800,000 new cases a year [1,2]. CRC is usually diagnosed in the elderly patient, with a median age at diagnosis of 71 years in the United States [3], with a growing incidence with advancing age, doubling every seven years in patients older than 50 years [3]. Many of these elderly patients would not be included in pertinent clinical trials given exclusion criteria of age or performance status, and some may not even undergo standard-of-care therapy. Frequently, next-generation gene sequencing (NGS) of their tumors is not even considered, all of which makes the management of this population very challenging [4].

This particular case highlights the use of a personalized approach and next-generation sequencing of this elderly patient's CRC tumor, leading to an exceptional and sustained response. Informed consent was obtained from the patient for this study.

## Case presentation

The patient is an 84-year-old female with a history of hypertension, hyperlipidemia, carotid artery stenosis, transient ischemic attack, and mild chronic kidney disease, who was in her usual state of health until May 2014 when she started to complain of worsening hematochezia. Colonoscopy was performed in four opportunities (due to persistent rectal bleeding without visible source), first in May 2014 and last in October 2014, which eventually revealed a fungating cecal mass and a rectosigmoid mass. Due to these findings, she underwent hand-assisted laparoscopic right hemicolectomy with excision of peritoneal, duodenal implants, liver wedge biopsy, and intraoperative ultrasound in October 2014. Surgical pathology reported high grade adenocarcinoma, pT4bN1bM1, 3/40 lymph nodes were involved. The duodenal nodule was positive and there was residual tumor left behind (R2 resection). An 18F-fluorodeoxyglucose (FDG) positron emission tomography (PET)/CT scan reported multiple FDG-avid hypermetabolic lesions within the liver, left-sided retroperitoneal lymphadenopathy, and anterior abdominal wall reactive changes. Due to her age and comorbidity, she was recommended to start 5-fluorouracil (5FU) and bevacizumab (Bev) palliative systemic chemotherapy.

She started 5FU and Bev from December 2014 through February 2015, for a total of six cycles, complicated by grade 2-3 diarrhea and volume depletion/AKI (acute kidney injury) requiring hospitalization, as well as unintended weight loss and decreased appetite due to nausea. She reported her diarrhea did not improve throughout her chemotherapy treatment, and only subsided once she discontinued it. Restaging PET/CT scan in March 2015 reported disease progression within the liver, retroperitoneum, anastomotic site, and distal rectal sigmoid colon. She was recommended hospice and best supportive care, but she declined it, and she was subsequently referred to the Abramson Cancer Center GI (Gastrointestinal) Oncology clinic for evaluation and treatment recommendations as a second opinion.

Next-generation sequencing testing of this patient’s tumor was ordered for genomic analysis in an attempt to determine potential therapeutic targets. This test was developed and its performance characteristics were determined by the University of Pennsylvania’s Center for Personalized Diagnostics Laboratory as required by the Clinical Laboratory Improvement Amendments (CLIA) 1988 regulations. Pursuant to the requirements of CLIA 1988, this laboratory has established and verified the test’s accuracy and precision. The methology of this 47-gene sequencing panel has been described elsewhere [5].

### Results

This analysis reported a frameshift mutation in adenomatous polyposis coli gene (APC) (allele frequency of 29.26%), a missense mutation in KRAS (allele frequency of 33.35%). Variants of uncertain significance were also identified in this patient’s sample: a missense variant in ERBB2 (allele frequency of 30.83%), and a missense variant in KDR at amino acid 961 converting the wild type residue, Arginine, to Tryptophan in 1068 reads out of a total 3780 sequence reads for an allele frequency of 28.25%. After multidisciplinary discussion at our molecular tumor board, it was determined that KRAS mutation made her ineligible for therapy with epidermal growth factor receptor (EGFR) inhibitors, however the KDR p.R961W c.2881C>T mutation that was noted as a variant of unknown significance (VUS) was discussed. The high allele frequency of this KDR mutation may deem the malignancy potentially sensitive to regorafenib, which is a tyrosine kinase inhibitor that targets KDR.

Regorafenib is approved by the Food and Drug Adminstration for the "treatment of patients with metastatic colorectal cancer (CRC) who have been previously treated with fluoropyrimidine-, oxaliplatin- and irinotecan-based chemotherapy, an anti-VEGF therapy, and, if KRAS wild-type, an anti-EGFR therapy" [6]. This patient's age and comorbidity did not make her eligible for combination therapy with either oxaliplatin or irinotecan, ergo it was decided to pursue treatment with regorafenib monotherapy based on its potential therapeutic effect targeting KDR. Given anticipated tolerance, she was started in March 2015 on regorafenib at the low dose of 80 mg daily for 21 days, however she developed grade 3 diarrhea and fatigue, which led to resume treatment at 40 mg once a day in May 2015, making the patient aware that such low dose may not have any therapeutic effect. A repeat NM PET/CT scan in May 2015 confirmed metastatic disease (largest lesion 27 x 17 mm hypodense lesion overlying the lateral aspect of right liver lobe with maximum SUV of 8.1). She tolerated this dose level much better, with overall symptomatic improvement, only complaining of grade 1 diarrhea managed with loperamide. Her carcinoembryonic antigen (CEA) was found elevated at 9.6 ng/mL in April 2015 prior to treatment, and it normalized at next follow-up, remaining within normal limits throughout her treatment.

**Figure 1:**
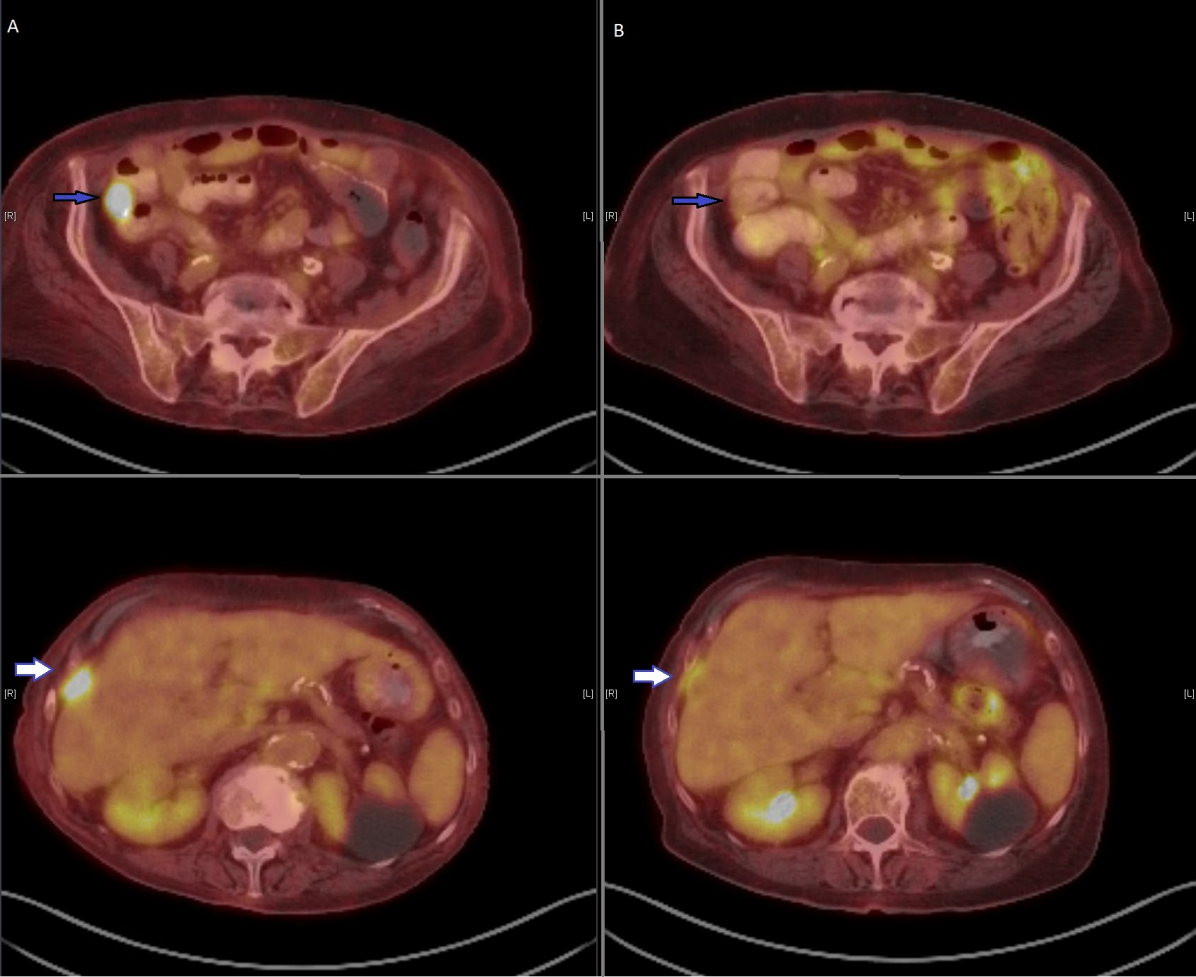
Response before and after regorafenib measured by PET/CT scan PET scan before (left panel A) and three months after low dose regorafenib (right panel B), showing improvement in right lower quadrant anastomotic soft tissue mass (blue arrow), and liver metastasis (white arrow)

Restaging NM PET/CT scan in August 2015 showed interval partial metabolic and radiologic response with resolved uptake in the lesion adjacent to the anastomotic site in the right lower quadrant, improvement in the hepatic metastases, abdominal and retroperitoneal lymph nodes, and rectosigmoid colon hypermetabolic lesions. No new hypermetabolic lesions were identified. (Figure 1)

Repeat restaging PET/CT scan in November 2015 reported continuous response to treatment with stable liver metastases, and improvement of retroperitoneal lymphadenopathy, without new FDG-avid metastatic disease. She continued therapy accordingly given no evidence of disease progression. After over nine months (39 weeks) of therapy, she remains almost completely asymptomatic, with the exception of some episodes of diarrhea. Her performance status improved dramatically since treatment started, and she was able to perform daily activities independently at home.

## Discussion

Colorectal cancer is predominantly a malignancy of the elderly; however, treatment options are limited due to the paucity of clinical data in this patient population, particularly regarding second-line and beyond therapies. In a review of 495 National Cancer Institute-sponsored cooperative group trials conducted from 1997 to 2000, elderly patients with CRC were significantly underrepresented in Phase II and III clinical trials [3,4]. Issues with performance status, anticipated tolerance and comorbidity are also significant barriers to enrollment. The most commonly used therapies are 5FU-based, followed by EGFR inhibitors (only if RAS wild type), anti-angiogenic agents (bevacizumab, aflibercept, ramucirumab), and regorafenib.

The Food and Drug Administration (FDA) approved regorafenib (Stivarga® tablets, made by Bayer HealthCare Pharmaceuticals, Inc.) for the "treatment of patients with metastatic colorectal cancer (mCRC) who have been previously treated with fluoropyrimidine-, oxaliplatin-, and irinotecan-based chemotherapy, with an anti-VEGF therapy, and, if KRAS wild type, with an anti-EGFR therapy" [6]. Regorafenib and its active metabolites inhibit multiple membrane-bound and intracellular kinases that are involved in normal cellular functions and pathologic processes, including those in the RET, VEGFR1, VEGFR2, VEGFR3, KIT, PDGFR-alpha, PDGFR-beta, FGFR1, FGFR2, TIE2, DDR2, Trk2A, Eph2A, RAF-1, BRAF, BRAFV600E, SAPK2, PTK5, and Abl pathways.

The approval was based on the CORRECT trial by Grothey et al [7], where 760 patients were randomized to receive regorafenib 160 mg (n=505) or placebo (n=255) for the first 3 weeks of each four-week cycle. The primary endpoint of overall survival was met at a preplanned interim analysis; median overall survival was 6.4 months in the regorafenib group versus 5.0 months in the placebo group (hazard ratio 0.77; 95% CI 0·64-0.94; one-sided p=0.0052). Treatment-related adverse events occurred in 465 patients (93%) assigned regorafenib and in 154 (61%) of those assigned placebo. The most common adverse events of grade three or higher related to regorafenib were hand-foot skin reaction (83 patients, 17%), fatigue (48, 10%), diarrhea (36, 7%), hypertension (36, 7%), and rash or desquamation (29, 6%). Mean duration of treatment was 2.8 months (SD 2.3; median 1.7, IQR 1.4–3.7) for the regorafenib group and 1.8 months (SD 1.2; median 1.6, IQR 1.3–1.7) for the placebo group. Median PFS was 1.9 months (IQR 1.6–3.9) in the regorafenib group and 1.7 months (1·4–1·9) in the placebo group.

Regarding the inclusion of elderly population, the oldest patient in the either cohort was 68 years old (median of 61). In this large Phase III trial of regorafenib, only five patients (1%) of 760 experienced a partial response (versus one patient, 0.4%, receiving placebo), and there was no NGS involved, only KRAS and BRAF were tested. This makes our 84-year-old patient in this report an exceptional responder in terms of overall survival, progression free survival (PFS), response rate, line of therapy, treatment duration, and tolerability at an anticipated less-than-therapeutic dose of regorafenib. 

The gene KDR encodes the protein VEGFR2, a receptor tyrosine kinase involved in angiogenesis [8]. The receptor binds to several related vascular endothelial growth factors (VEGF) as ligands, and via its downstream signaling pathway serves as a crucial component of the process of physiologic angiogenesis. VEGFR2 is also involved in pathologic angiogenesis and has been found to be upregulated in endothelial cells in a wide variety of solid tumors [8,9]. Moreover, VEGFR2 levels have been shown to be associated with increased vascularity and metastatic potential in colorectal cancer [10].

While VEGFR2 overexpression in human malignancies is well described, the oncogenic role of specific mutations in KDR* *is less well-defined. Regarding colorectal cancer (CRC) in particular, the presence of mutations in KDR, along with those in several other tyrosine kinases, have long been described in CRC [11]. Several genetic studies have sought to identify predictive markers of efficacy of antiangiogenic therapies, such as regorafenib [12]. Unfortunately, such genetic markers, along with other potential biomarkers, have not been readily identified thus far [13]. For instance, regarding anti-VEGF therapy with the monoclonal antibody bevacizumab, clinical benefits have largely been found to be independent of KRAS and BRAF mutational status. Similarly, regarding the use of regorafenib in particular, a retrospective analysis of the CORRECT cohort demonstrated an improvement in progression-survival among patients, regardless of KRAS and PIK3CA-mutational status [13]. Nevertheless, KDR has not been assessed prospectively as a predictive biomarker of response to regorafenib. KDR p.R961W c.2881C>T mutation has been reported twice in COSMIC [14] but there has been no functional data reported, and it is reported as a variant of unknown significance (VUS). The mutation occurs in the tyrosine kinase domain of VEGFR2, making it a potential and biologically plausible target for regorafenib. 

## Conclusions

This case is remarkable not only for the durable and significant symptomatic and tumor marker response in this elderly patient with treatment-refractory KRAS mutant CRC, but also for demonstrating an exceptional responder case in terms of overall survival, PFS, line of therapy, response rate, treatment duration, and tolerability at an anticipated less-than-therapeutic dose of regorafenib. The use of NGS technology and a personalized approach led to the potential identification of a predictive biomarker of response.

Hereby we hypothesize KDR mutation as a novel candidate predictive biomarker to exceptional response to regorafenib in metastatic colorectal cancer. To our knowledge, this is the first reported case of the potential association between KDR mutation and regorafenib use for the successful management of a patient with advanced CRC, leading to what is considered an exceptional response. As we advance our knowledge of the implications of less commonly described cancer-related gene aberrations, many VUS mutations would become candidates for targeting with novel therapeutic developments, and they will provide the foundation for 'N of 1' and 'basket' trials such as the NCI-Molecular Analysis for Therapy Choice (NCI-MATCH) and Targeted Agent and Profiling Utilization Registry (TAPUR) [15]. Further prospective studies based on this preliminary data are warranted. 

## Appendices

Next-generation sequencing testing of this patient’s tumor was ordered for genomic analysis in an attempt to determine potential therapeutic targets. This test was developed and its performance characteristics were determined by the University of Pennsylvania’s Center for Personalized Diagnostics Laboratory as required by the Clinical Laboratory Improvement Amendments (CLIA) 1988 regulations. Pursuant to the requirements of CLIA 1988, this laboratory has established and verified the test’s accuracy and precision.

Methodology

Genomic DNA was extracted from the submitted specimen (paraffin-embedded tissue block from initial diagnostic liver biopsy) according to manufacturer’s instructions (Qiagen, Inc.). Targeted analysis for mutations in the regions specified in this testing panel was achieved by the enrichment of those genomic loci using the Illumina Truseq Amplicon Assay (Illumina, Inc ®). Sequencing of enriched libraries was performed on the Illumina MiSeq platform using multiplexed, paired-end reads with Version 2 chemistry. Analysis and interpretation utilized a customized bioinformatics process. All variants listed are with reference to the hg19 Genome build. Variants are reported according to Human Genome Variation Society (HGVS) nomenclature and classified into three categories: pathogenic, variants of uncertain significance, and benign. Categorization of variants was dependent upon literature review, variant existence in a variety of publically available databases, including the Single Nucleotide Polymorphism Database (dbSNP), the Catalogue of Somatic Mutations in Cancer (COSMIC), and the 1,000 genome project. Variants classified as benign are not listed in the final report.

This assay detects single nucleotide variants and small insertions or deletions (indels). Large or complex indels, inversions, translocations, gene amplifications, copy number changes or other complex genomic mutations may not be detected by this assay. Variants existing outside the target regions cannot be detected. Only variants in the exonic regions of the gene panel are reported. This assay does not determine variant causality, or whether a variant is inherited or somatically acquired. This assay will detect variants representing at least 10% of the total sequence reads at a given genomic position. During test validation, the choice of parameters was optimized to maximize sensitivity and specificity.

Solid tumour-sequencing panel included sequence analysis of 47 genes (ABL1, AKT1, ALK, APC, ATM, BRAF, CDH1, CSF1R, CTNNB1, EGFR, ERBB2, ERBB4, FBXW7, FGFR1, FGFR2, FGFR3, FLT3, GNA11, GNAQ, GNAS, HNF1A, HRAS, IDH1, JAK2, JAK3, KDR, KIT, KRAS, MET, MLH1, MPL, NOTCH1, NPM1, NRAS, PDGFRA, PIK3CA, PTEN, PTPN11, RB1, RET, SMAD4, SMARCB1, SMO, SRC, STK11, TP53, and VHL). Genomic regions not meeting criteria for variant calling include the genomic loci for which the depth of coverage did not meet our minimum criteria of 250 reads at all positions within those loci. Since the sensitivity and specificity of our assay were determined by achieving a minimum depth of coverage of 250 reads, regions not meeting this criterion cannot be guaranteed to be mutation negative. The depth of coverage is dependent upon the starting amount of DNA for a given region that could be affected by underlying chromosomal copy number changes. Since the genomic loci targeted in this assay are enriched using loci-specific primers, patient variants existing within those primer sequences may also cause a region to fail enrichment and therefore not achieve 250x coverage. The design of the panel was based on the literature at the time of development; either the full length of a gene or the mutational hot spots of a gene are targeted.
